# A new transformation of cone responses to opponent color responses

**DOI:** 10.3758/s13414-020-02216-7

**Published:** 2021-01-06

**Authors:** Ralph W. Pridmore

**Affiliations:** Central Houses Pty Ltd, 8C Rothwell Rd, Turramurra, Sydney, NSW 2074 Australia

**Keywords:** Color and Light: Color, Neural mechanisms

## Abstract

It is widely agreed that the color vision process moves quickly from cone receptors to opponent color cells in the retina and lateral geniculate nucleus. Many workers have proposed the transformation or coding of long, medium, short (*LMS*) cone responses to *r − g, y − b* opponent color chromatic responses (unique hues) on the following basis: That *L*, *M*, *S* cones represent Red, Green, and Blue hues, with Yellow represented by (*L* + *M*), while *r − g* and *y − b* represent the opponent pairs of unique hues. The traditional coding from cones to opponent colors is that *L − M* gives *r − g*, while (*L* + *M*) − *S* gives *y − b*. This convention is open to several criticisms, and a new coding is required. A literature search produced 16 studies of cone responses *LMS* and 15 studies of spectral (i.e., *ygb*) opponent color chromatic responses, in terms of response wavelength peaks. Comparative analysis of the two sets of studies shows the means are almost identical (within 3 nm; i.e., *L = y, M = g, S = b*). Further, the response curves of *LMS* are very similar shapes to *ygb*. In sum, each set can directly transform to the other on this proposed coding: (*S* + *L*) − *M* gives *r − g*, while *L − S* gives *y − b*. This coding activates neural operations in the cardinal directions *r − g* and *y − b*.

Color vision theory comprises the two theories of trichromacy and opponent colors. It is generally accepted, therefore, that the color vision process consists of a minimum of two stages, commencing with three cone receptors and moving quickly (but not necessarily directly) to opponent color cells in the retina and lateral geniculate nucleus (LGN). For example, Buchsbaum and Gottschalk (1983) argue that efficient information transmission requires a prompt transformation of the three cone mechanisms into an achromatic and two opponent chromatic channels. Many others, such as Svaetichin (1956) and De Valois (1965), similarly show that signals from different cones are combined in an opponent manner in the second stage. The opponent or interactive nature of the second stage is widely supported by various workers, including Hurvich and Jameson’s (Jameson & Hurvich, 1955; Hurvich & Jameson, 1955; Hurvich & Jameson, 1957) hue cancellation experiments and other evidence for mixtures of chromatic signals (Boynton, Ikeda, & Stiles, 1964; Mollon & Polden, 1975; Krauskopf, 1973).

To this end, most workers (e.g., Conway, 2009; Wiesel & Hubel, 1966; Dacey & Lee, 1994; Reid & Shapley, 2002; Field et al., 2007; Foster & Amano, 2019) have proposed the transformation or coding of *LMS* cone responses (see Fig. 1) to *r − g, y − b* opponent color chromatic responses (see Fig. 2) on the following basis: that *L*, *M*, *S* cones represent Red, Green, and Blue hues (with Yellow represented by [*L* + *M*]), while *r − g* and *y − b* represent opponent pairs of unique hues. The widely accepted traditional coding from cones to opponent colors is that *L − M* gives *r − g*, while (*L* + *M*) − *S* gives *y − b*. This convention is open to several criticisms, including (a) that *L* alone cannot produce unique *r,* because the latter is not spectral (all spectral reds are yellowish) but a nonspectral hue, therefore requiring input from a short and a long wavelength, *S* + *L*; (b) that the wavelength peaks of *L* and *M* cones are too close (at about 565 and 535 nm) to represent opponent colors/unique hues *r* and *g*; and, further, (c) since those *L* and *M* cones cannot represent opponent colors *r* and *g*, then *L* + *M* cannot represent unique *y,* from (*r* + *g*). Thus, the traditional coding has problems with producing unique hues Red, Green, and Yellow (i.e., three of the total four unique hues). Clearly, an improved form of coding or transformation from cone responses to opponent color responses is required. To find such a transformation is this paper’s aim.

**Fig. 1:**
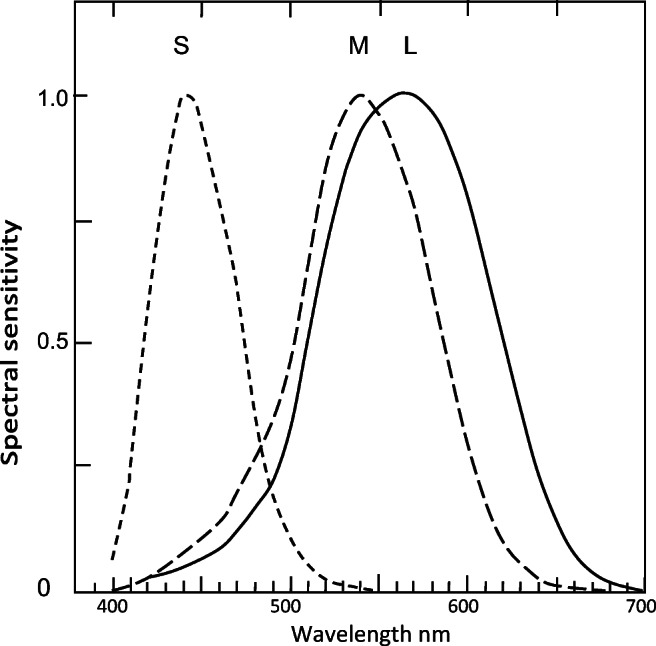
Normalized *SML* cone sensitivity functions, drawn from Stockman and Sharpe (2000) log values recalculated to arithmetic/linear (*y*-axis). This is Stage 1 of traditional theory agreed by all models of color vision. These curves and peaks are closely similar to other experimental data

**Fig. 2:**
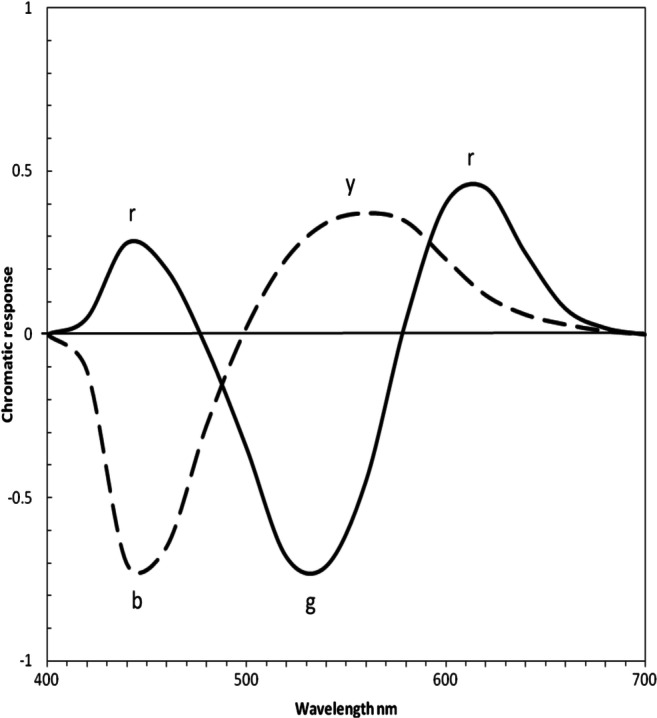
Opponent color chromatic responses *y − b* (dashed) and *r −* g *(*solid line), redrawn from Hurvich (1981). This is the final stage of traditional color vision theory

Note that unique hues and opponent color chromatic responses are here denoted by *r, y, g, b* as in convention. The Red Green Blue peaks of trichromatic functions are denoted by *RGB*.

## Methods

Required is a comprehensive database of cone sensitivities (also known as cone fundamentals) and opponent color chromatic responses gathered from all or most published scientific studies. A literature search (dated 25 Feb. 2019, per Macquarie University, Sydney, subscription to Web of Science) utilized Web of Science, Core Collection, with search terms “cone sensitivities” or “opponent color chromatic responses” as “title.” This produced 118 publications. Manual search of 20 books and the archives of 13 major journals publishing color vision, physiological optics, and color science, produced another 33 publications, totaling 151. Of these, five were removed as duplicates, and a further 111 removed as unrelated to cones and opponent colors or as abstracts and conference proceedings lacking essential detail. This left 35 full text publications, of which 4 were removed as measuring enucleated cones rather than in vivo.

The 31 remaining studies (16 for cone sensitivities, 15 for opponent color responses) are shown in Table 1 and were applied to meta-analysis of the question, How closely similar are the wavelength peaks of cone sensitivities and spectral opponent color responses? The mean wavelength peaks of these two types of functions were compared, curve shapes were formulated (see Equations 1–3) and compared, and correlation coefficients were calculated (see Table 2). Data from each study were treated without bias by (a) listing only previously published studies, taking the peer review process to guarantee a minimum standard of study, (b) taking from each study the same parameters (i.e., the function’s trio of wavelength maxima), and (c) defining which statistical methods (i.e., weighting and arithmetical means) are to be employed for each study.

**Table 1:** Wavelength maxima for cone sensitivities and opponent color chromatic responses for various experimental data sets

Human cone spectral sensitivities from psychophysical and experimental data				Spectral opponent color chromatic responses from hue cancellation experiments			
*S*	*M*	*L*	References	*b*	*g*	*y*	References
440	540	565	Smith and Pokorny (1975)	435	530	550	Jameson and Hurvich (1955)
445	540	560	Judd and Wyszecki (1975)	445	530	560	Hurvich (1981)
444	527	571	Estevez (1979)	440	530	555	Romeskie (1976)
450	540	560	Wyszecki and Stiles (1982a)	455	527	582	Werner and Wooten (1979a)
440	540	565	Wyszecki and Stiles (1982b)	440	530	575	Werner and Wooten (1979b)
444	530	571	Wyszecki and Stiles (1982c)	440	540	560	Fuld (1991)
438	533	564	Dowling (1987)	440	525	560	Kulp and Fuld (1995)
440	530	560	Stockman, MacLeod, and Johnson (1993)	440	530	560	Yaguchi and Ikeda (1982)
440	540	565	Stockman and Sharpe (2000)	440	535	575	Ingling and Tsou (1977)
445	535	570	Vos, Estevez, and Walraven (1990)	447	535	565	Takahashi, Ejima, and Akita# (1985)
440	532	568	Smith, Pokorny, and Zaidi (1983)	445	530	565	Takahashi et al.† (1985)
445	542	570	Hunt and Pointer (2011)	445	530	564	Takahashi et al.* (1985)
440	520	560	Foster and Snelgar (1983)	445	535	570	Takahashi et al. L (1985)
450	525	555	Brown and Wald (1964)	445	535	565	Takahashi et al. T (1985)
430	540	570	Wald (1964)	450	530	560	Gegenfurtner (2003)
445	540	585	Stiles (1964)	*443.5*	*531.5*	*564.4*	*Means*
*442.3*	*534.6*	*565.8*	*Means*				

*Note.* Values in Table 1 are experimental data or derived therefrom, listing wavelength peaks of cones and spectral opponent colors, showing the two sets are closely similar. Under References column, only the first author is listed. Wyszecki references (Wyszecki & Stiles, 1982a; Wyszecki & Stiles, 1982b; Wyszecki & Stiles, 1982c) (see Reference list) denote, respectively, Wyszecki and Stiles (1967) Konig-type fundamentals, Vos and Walraven (1978) fundamentals, and Stiles (1953, 1959) field sensitivities. From Takahashi et al. (1985), three troland levels are shown for 8,700 K color temperature and two subjects: # denotes 50 td, † is 500 td, * is 5,000 td. For 5,200 K and one subject, L denotes 50 td, T is 500 td. All data sets are given Weight 2 (see main text), except the Brown, Wald, and Stiles cone data (Brown & Wald, 1964; Wald, 1964; Stiles, 1964) are weighted 1 rather than 2 since they derive from the less reliable test sensitivities method

**Table 2:** Pearson correlation coefficients between sets of curves or sets of wavelength maxima

Correlation coefficients			
Correlation between unitized cone response curves and unitized chromatic response curves:			
*S* and *b*: 0.989	*M* and *g*: 0.870	*L* and *y*: 0.986	Mean: 0.948
Between predicted curves (Eqns 1–3) and cone response curves:			
Eqn 1 and *S*: 0.981	Eqn 2 and *M*: 0.985	Eqn 3 and *L*: 0.995	Mean: 0.987
Between predicted curves (Eqns 1–3) and chromatic response curves:			
Eqn 1 and *b*: 0.985	Eqn 2 and *g*: 0.931	Eqn 3 and *y*: 0.986	Mean 0.967
Between *SML* and *bgy* sets of wavelength maxima (see Table 1):			
0.9996			

*Note.* Data are calculated at 5-nm intervals for unitized curves *SML* and *bgy* from Refs (Stockman & Sharpe, 2000; Hurvich, 1981)

## Results

Table 1 shows values in column *S* of the cones group are very similar to those in column *b* of opponent colors group, and similarly for columns *M* and *g*, and columns *L* and *y*. Arithmetical means of the two groups are extremely close at 442.3, 534.6, and 565.8 nm for cones, and 443.5, 531.5, and 564.4 nm for opponent colors, differing by 3.2 nm at most. Therefore, from close agreement in the wavelengths of the peaks, we can propose the hypothesis *L = y, M = g, S = b*.

In Table 1, weighting method is used to indicate reliability of data as follows. Top weighting is Weight 2, given for all properly conducted studies and independent of the number of subjects since one cannot know the number of subjects for those studies which are secondary sources. Such secondary sources are widely regarded in the literature as sometimes more accurate to human vision than the original experiments. Weight 1 is given to Refs (Brown & Wald, 1964; Wald, 1964; Stiles, 1964) due to the unreliable method of test sensitivities no longer used. Rather surprisingly, a Stiles study is in that small group, but he only submitted his test sensitivities data for publication after invitation by Wald. Stiles and other researchers generally used the more reliable field sensitivities method still in use today.

The standard deviations to the Table 1 means for *S, M*, and *L* are 4.9, 6.7, and 7.0 nm, respectively; and the standard deviations to the means for *b, g*, and *y* are 5.0, 3.8, and 8.3 nm, respectively. These standard deviations indicate the maximum discrepancy of 3.2 nm (mentioned above) is relatively small.

**Fig. 3:**
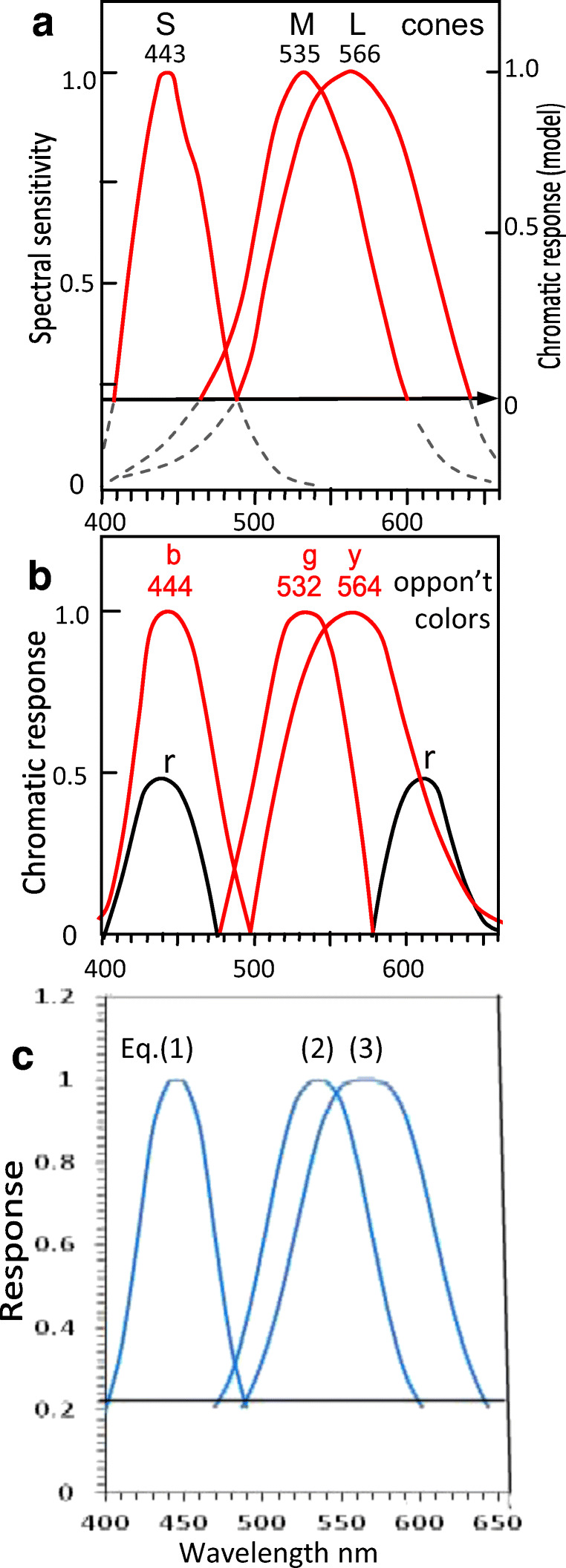
Response curves with wavelength peaks labelled per means of Table 1, rounded to nearest integer. All curves normalized to 1.0 response. **a** *SML* cone sensitivities (from Fig. 1). Only the curves (in red) above arrowed line are compared with opponent color curves (red curves in Fig. 3b) in calculating correlation coefficients. Intersection of *S* and *L* curves (arrowed line) shows level of zero potential chromatic response (right-hand *y*-axis) in the same manner as intersection of *b* and *y* curves in Fig. 2 or Fig. 3b, giving a response scale equivalent to opponent colors in Fig. 3b. **b** The three spectral opponent color chromatic response curves (red lines) *b, g, y,* as in Fig. 2, but all normalized to positive 1.0*.* Black curves represent short and long wavelength components of opponent color chromatic response *r*. **c** Predicted curves 1–3 from curve-fitting Equations 1–3 in main text (e.g., curve 1 represents curves *S* and *b* equally well)

Figure 3 shows all curves normalized to 1.0. The curves of *L*, *M*, and *S* are very similar to those for *y*, *g*, and *b,* respectively, as shown by Equations 1–3 and correlation coefficients below, indicating *LMS* and *ygb* curves are practically equivalent. Figure 3a shows the parts of the cone curves (in red, above the horizontal cutoff line) to be compared with the opponent color curves. The intersection of *S* and *L* curves (arrowed line) shows the level of zero potential chromatic response (right-hand *y*-axis) in the same manner as the intersection of *b* and *y* curves in Fig. 2 or Fig. 3b. Figure 3c shows the curves plotted from Equations 1–3.

Equation 1 represents with equal accuracy the two function curves for cone *S* and opponent color chromatic response *b*; similarly, Equation 2 represents cone *M* and opponent color *g*, and Equation 3 represents cone *L* and opponent color *y*.

1 $$ 1/\upalpha ={\left[\uplambda \hbox{--} {\uplambda}_{\mathrm{max}}/28.5\right]}^{3.1}+1 $$

2 $$ 1/\upalpha ={\left[\uplambda \hbox{--} {\uplambda}_{\mathrm{max}}/41\right]}^{2.8}+1 $$

3 $$ 1/\upalpha ={\left[\uplambda \hbox{--} {\uplambda}_{\mathrm{max}}/52\right]}^{3.3}+1 $$

where λ represents wavelength on the *x*-axis of Fig. 3c, λ_max_ represents the curve’s wavelength peak, and α represents response amplitude (spectral sensitivity on left *y*-axis).

**Fig. 4:**
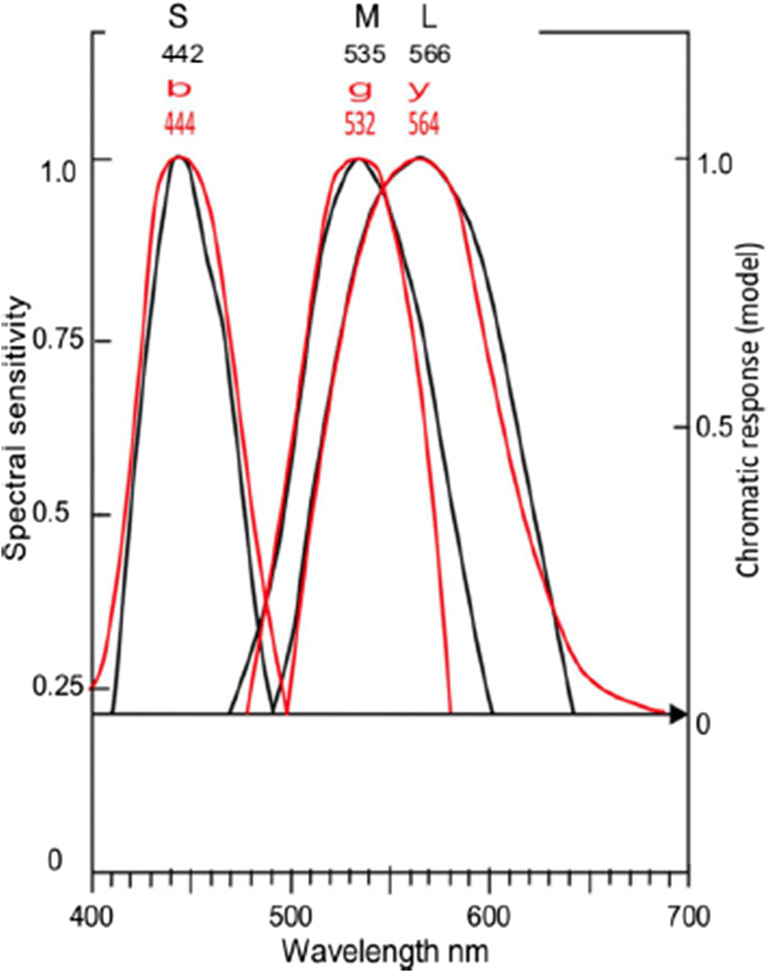
Cone sensitivities *SML* (in black) with spectral opponent color chromatic responses *bgy* (in red) copied from Fig. 3 and overlaid on the former. The 0 response level derives from the arrowed line marking the level where *S* and *L* curves intersect. The opponent color curves (red) are shifted laterally so their wavelength maxima align with wavelength maxima of the cones (per Table 1) as labeled

The peak wavelengths and shape of the function curves (see Fig. 3c) are much the same for each cone and its associated opponent color, as quantified by correlation coefficients in Table 2. For example, both curves *S* and *b* are the narrowest curves, and both *L* and *y* are the broadest curves. Correlation coefficient between the associated pairs (e.g., *S* and *b*) is a mean 0.948.

In Fig. 4, cone curves *SML* and spectral opponent color curves *bgy* (taken from Fig. 3) are overlaid to show the close similarity of the two sets of curves. The only notable difference is between the long wavelengths of the *M* and *g* pair of curves.

This comparison (see Tables 1 and 2, and Figs. 3 and 4) of the cone responses and spectral opponent color chromatic responses is more comprehensive than attempted previously and consequently finds relationships previously unknown in the literature.

## Proposed coding

The above comparative analysis of the two sets of studies shows that the response curves for *LMS* are practically identical to those for *ygb*. As a general guide, the similarity of stages, or the functions that characterize those stages, indicate the proximity or sequence of those stages in a neurocognitive process such as the visual process. From the close degree of similarity described above, one may deduce that one group (the *LMS* cones as Stage 1) is the direct precursor of the other (*ygb* unique hues as Stage 2, plus the *r* unique hue formed neurally from input of a short and a long wavelength *S* + *L*). Hence, each group can be directly transformed to the other on this proposed coding: (*S* + *L*) − *M* gives *r − g*, while *L* − *S* gives *y − b*. Each opponent pair is a pair of unique hues.

This proposed coding avoids the criticisms of traditional coding mentioned above: (a) that *L* cannot produce unique *r* because the latter is not a spectral but a nonspectral hue, therefore requiring input from a short and a long wavelength, *S* + *L*; (b) that the wavelength peaks of *L* and *M* cones are too close (at about 566 and 535 nm) to represent opponent colors/unique hues *r* and *g*; and further, (c) since those *L* and *M* cones cannot represent opponent colors *r* and *g*, then *L* + *M* cannot represent unique *y* (from *r* + *g*).

The different transformations may be illustrated by matrix equations, below. The transformation of cone responses to opponent color chromatic responses in previous literature is that given in Equation 4, while the one proposed in the current paper is given in Equation 5:

4 $$ \left(\begin{array}{c}r-g\\ {}y-b\end{array}\right)=\left(\begin{array}{c}1\kern1em -1\kern1.5em 0\\ {}1\kern2em 1\kern0.75em -1\end{array}\right)\ \left(\begin{array}{c}L\\ {}M\\ {}S\end{array}\right) $$

5 $$ \left(\begin{array}{c}r-g\\ {}y-b\end{array}\right)=\left(\begin{array}{c}1\kern1em -1\kern1.5em 1\\ {}1\kern2em 0\kern0.75em -1\end{array}\right)\ \left(\begin{array}{c}L\\ {}M\\ {}S\end{array}\right) $$

## Discussion

The short and long wavelengths *S* + *L* forming the *r* unique hue presumably approximate 442 and 613 nm, the optimal pair for additive mixture of all nonspectral hues (Pridmore, 1978; Pridmore, 1980). This proposed coding is equivalent to Jameson and Hurvich’s (1972) final version of coding cones to opponent colors, where the coefficient amount (less than 0.1) ascribed to cone *M* (in the coding *L* + *M* ≡ *Y*) is trivial and may be ignored, leaving *L* ≡ *y*. This proposed coding activates neural operations in the cardinal directions *r − g* and *y − b*, as does the original or traditional coding.

The demonstrated similarity between cone responses and opponent color chromatic responses places a higher significance in the cone responses than previously thought, since the cones may now be regarded as the precursors of the spectral opponent colors and unique hues Yellow, Green, and Blue. Unique Red is later formed neurally by additive mixture of a short and long wavelength (as in some conventional color-coding, e.g., Jameson & Hurvich (1972)). It is concluded that this simplified transformation represents an improvement to conventional vision theory.

Though not central to this paper, the location and derivation of the *RGB*-peaked functions typical of trichromatic color vision (such as color matching, spectral sensitivity, wavelength discrimination, and saturation, with peaks about 605, 535, 445 nm) are of interest. They may be located in either of two stages suggested in the literature. Traditional theory claims Stage 1 contains all aspects of trichromacy since *RGB*-peaked functions can be linearly transformed (by a process of three-matrix algebra) to/from the *LMS* cone curves. As a matter of fact, the *LMS* curves or the *RGB* color matching curves can be converted linearly to an infinite number of curve sets, which proves nothing relevant. The major problem is the very different peak wavelength of *L* (about 565 nm) relative to *r* (about 605 nm). This begs the question of whether the *L* peak can represent an either/or condition (565 nm for the cone or 605 nm for the *RGB* function), or whether it can in logic represent both values at one time or in the one stage (i.e., Stage 1). Logic (and Occam’s Razor) suggests not.

Alternatively, Pridmore (2011) holds that Stage 1 represents only the *LMS* cone receptors, with the opponent color responses *y − b* and *r* − *g* located in Stage 2, and the *RGB* functions of trichromatic vision located in the final Stage 3, transformed from opponent color responses by special summation. The fact that simple addition (i.e., special summation) converts opponent color curves to the *RGB* curves of trichromatic vision indicates the latter relate directly to the former (*y − b, r − g*) rather than to the *LMS* curves. Further, normal trichromatic vision and its *RGB* functions is logically located as the end result of the visual process rather than in the first stage with the cones.

This research relates to the goals of recently approved CIE Technical Committee 1-98 entitled A Roadmap Toward Basing CIE Colorimetry on Cone Fundamentals.
